# High variability of perezone content in rhizomes of *Acourtia cordata* wild plants, environmental factors related, and proteomic analysis

**DOI:** 10.7717/peerj.16136

**Published:** 2023-11-15

**Authors:** Ma del Carmen García Méndez, Sergio Encarnación-Guevara, Ángel Gabriel Martínez Batallar, Leopoldo Gómez-Caudillo, Roque Bru-Martínez, Ascensión Martínez Márquez, Susana Selles Marchart, Efraín Tovar-Sánchez, Laura Álvarez-Berber, Silvia Marquina Bahena, Irene Perea-Arango, José de Jesús Arellano-García

**Affiliations:** 1 Centro de Investigación en Biotecnología, Universidad Autónoma del Estado de Morelos, Cuernavaca, Morelos, México; 2 Centro de Ciencias Genómicas, Universidad Nacional Autónoma de México, Cuernavaca, Morelos, México; 3 Departamento de Agroquímica y Bioquímica, Facultad de Ciencias, Universidad de Alicante, Alicante, Spain; 4 Instituto de Investigación Sanitaria y Biomédica de Alicante, Instituto de Investigación Sanitaria y Biomédica de Alicante, Alicante, Spain; 5 Centro de Investigación en Biodiversidad y Conservación, Universidad Autónoma del Estado de Morelos, Cuernavaca, Morelos, Mexico; 6 Centro de Investigaciones Químicas, Universidad Autónoma del Estado de Morelos, Cuernavaca, Morelos, Mexico

**Keywords:** Perezone, Secondary metabolites, *Acourtia cordata*, Plant proteome, Edaphic factors, Rhizomes

## Abstract

With the aim of exploring the source of the high variability observed in the production of perezone, in *Acourtia cordata* wild plants, we analyze the influence of soil parameters and phenotypic characteristics on its perezone content. Perezone is a sesquiterpene quinone responsible for several pharmacological effects and the *A*. *cordata* plants are the natural source of this metabolite. The chemistry of perezone has been widely studied, however, no studies exist related to its production under natural conditions, nor to its biosynthesis and the environmental factors that affect the yield of this compound in wild plants. We also used a proteomic approach to detect differentially expressed proteins in wild plant rhizomes and compare the profiles of high *vs*. low perezone-producing plants. Our results show that in perezone-producing rhizomes, the presence of high concentrations of this compound could result from a positive response to the effects of some edaphic factors, such as total phosphorus (P_t_), total nitrogen (N_t_), ammonium (NH_4_), and organic matter (O. M.), but could also be due to a negative response to the soil pH value. Additionally, we identified 616 differentially expressed proteins between high and low perezone producers. According to the functional annotation of this comparison, the upregulated proteins were grouped in valine biosynthesis, breakdown of leucine and isoleucine, and secondary metabolism such as terpenoid biosynthesis. Downregulated proteins were grouped in basal metabolism processes, such as pyruvate and purine metabolism and glycolysis/gluconeogenesis. Our results suggest that soil parameters can impact the content of perezone in wild plants. Furthermore, we used proteomic resources to obtain data on the pathways expressed when *A. cordata* plants produce high and low concentrations of perezone. These data may be useful to further explore the possible relationship between perezone production and abiotic or biotic factors and the molecular mechanisms related to high and low perezone production.

## Introduction

The plant species *Acourtia cordata* (Cerv.) B. L. Turner (*Asteraceae*) is commonly known as the “Buzzard herb” and has been used in traditional Mexican medicine as an infusion possessing anthelmintic, laxative, and diuretic properties (Flores y Troncoso & de Asís, 1982). The genus *Acourtia* comprises approximately 80 species, most of which are endemic to Mexico (Villaseñor, 2016). The rhizomes of several species of this genus produce many compounds of terpenic origin, including the sesquiterpene *p*-benzoquinone perezone, which is highly soluble in n-hexane and can be easily extracted and purified with this solvent. The perezone was the first substance isolated and purified from the New World (in 1852), and its chemistry has been widely studied. It is known to possess quinone-like properties; it oxidizes easily and reduces spontaneously (Joseph-Nathan, González & Rodríguez, 1972; Joseph-Nathan & Santillan, 1989). Recently, the importance of perezone and more than 100 related compounds has been reviewed, as well as their biological activities, which have been highlighted (Escobedo-González et al., 2022). Interestingly, they report several plant species that not belonging to the genus *Acourtia*, produce perezone and/or related compounds.

In addition, several pharmacological activities have been reported for perezone and its derivatives: hypoglycemic activity, inhibition of platelet aggregation, induction of contractile response in intestinal smooth muscle, and protection against the deleterious effects of ischemia and reperfusion (Alarcon-Aguilar et al., 1997; Peña et al., 2001; Garcia et al., 1995; Tellez et al., 1999). The antifeeding effects of perezone derivatives against larvae of agriculturally important insect pests and their phytotoxic activity have also been reported (Burgueño-Tapia et al., 2008).

Some reports on the biotechnological production of perezone have shown that callus, hairy roots, and *in vitro* cultured plantlets of some *Acourtia* species produce and accumulate lower amounts of perezone than the rhizomes of native plants (Arellano et al., 1996; Gómez-Serrano, Cristiani-Urbina & Villegas-Garrido, 2010, 2012). This biosynthesis and accumulation of secondary metabolites (SMs) can vary in a plant species due to genotypic differences among individuals growing in the same area. The variation may also be due to different environmental conditions in the places where each plant grows. Such environmental factors include altitude, latitude, precipitation, temperature, humidity, soil, sun radiation, *etc*. (Liu et al., 2015; Moreira et al., 2018; Filippi et al., 2021). The quantitative and qualitative SM variation in plant species occurs among individuals, tissue types, ontogenetic stages, plant age and size (Koricheva & Barton, 2012; Lattanzio et al., 2009; Andrew et al., 2007). Therefore, the population age and structure can contribute to SM variation (Moore & Foley, 2005). Edaphic parameters also contribute to the expression of different biochemical characteristics in plants (Hamilton et al., 2001). Phosphorous (P) and nitrogen (N) are the two most limiting elements in plants since they alter diverse physiological and biochemical processes (Pradhan et al., 2017; Ramakrishna & Ravishankar, 2011). N and P can increase the accumulation of carbohydrates for plant growth and photosynthetic rates (Bassi, Menossi & Mattiello, 2018). They can also modify the biosynthesis of carbon-based SMs, such as phenolic acids, flavonoids, tannins, and terpenes (Ormeño & Fernandez, 2012).

Proteomics is an important tool to describe diverse processes in the plant kingdom, such as morphogenetic, physiological, and biochemical changes during the development of tissues and organs (Di Michele et al., 2006; Tian et al., 2009; Mohamad et al., 2011; Takáč, Pechan & Šamaj, 2011; Martínez-Márquez et al., 2013; Martínez-Esteso et al., 2014). In addition, proteomics can facilitate the analysis of proteins related to different biotic and abiotic stresses (Sergeant & Renaut, 2010; Fernandez-Garcia et al., 2011; Trupiano et al., 2012; Aghaei & Komatsu, 2013; Ghosh & Xu, 2014; Fang et al., 2015; Garcia de la Garma et al., 2015; Chmielewska et al., 2016; Martinez-Esteso et al., 2016). Furthermore, proteomics enables the study of proteins engaged in biosynthetic pathways that lead to the biosynthesis of SMs in medicinal plants (Senthil et al., 2011; Martinez-Esteso et al., 2011; Champagne et al., 2012; Bhattacharyya et al., 2012; Ma et al., 2013; Sud, Chauhan & Tandon, 2014; Bryant et al., 2015; Martínez-Esteso et al., 2015).

Due to liquid chromatography–mass spectrometry and bioinformatics clear progress in plant proteomics has been seen (Baginsky, 2008). Based on this technology, many types of studies have been conducted for the analysis of complex mixtures of proteins obtained from tissues of model and non-model plant species (Bayer et al., 2006; Armengaud et al., 2014). Although there are many proteomic studies with different approaches in model plants, there are only a few in non-model plants, mostly in medicinal plants (Oldham et al., 2010).

The plants of *A. cordata* are a natural source of several bioactive compounds responsible for different pharmacological effects as perezone. Nevertheless, there are no reports to date on the probable effect or relation of the edaphic factors impacting production of sesquiterpenes such as perezone. Also, there are no reports on the identification, location, and characterization of proteins involved in perezone biosynthesis.

In a preliminary study carried out in the Alarcon locality, we found a high variability of perezone content between the rhizomes of 10 individual wild plants of *A. hebeclada* synonymous with *A. cordata*, ranging from 0.00–0.29 mg/g dry weight (García-Méndez et al., 2016). Hence, the aim of the present study was to analyze the relationship between the high variability of perezone content in the rhizomes of *A. cordata* wild plants and rhizospheric soil parameters and plant macromorphological characteristics, as well as provide information on the proteins differentially expressed in rhizomes of *A. cordata* with different concentrations of perezone. In this sense, we hypothesized that soil components, macromorphological characteristics, and differences in protein expression could be related to differences in perezone content between high and low producers in *Acourtia cordata* wild plants.

## Materials and Methods

### Plant material

This study used the rhizomes of 60 wild plants of *A. cordata* from: Chamilpa, Alarcon, and Felipe Neri. These are located inside the ecologically protected area known as “Chichinautzin”, in the state of Morelos located at 2,309 m.a.s.l. and 18°45′0″N, 99°4′0″W (Vega Guzmán, López-García & Manzo Delgado, 2008). All the plant material collected in the field was carried out according to the protocols of the Universidad Autónoma del Estado de Morelos/Corredor Biológico Chichinautzin. Each rhizome sample was immediately placed in a plastic bag and stored on ice for transport to the laboratory (Fig. S1). Once in the laboratory, each sample was divided in two parts: one part was used for perezone quantification and the other part was carefully washed and stored at −80 °C. A plant specimen from each locality (voucher numbers 34664, 34665, and 34666) was deposited at the HUMO-Herbarium of the Biodiversity and Conservation Research Center of the Autonomous University of the State of Morelos, Mexico (Fig. S2). The macromorphological characteristics of each sampled plant (basal diameter, number of branches, total individual height, and shrub cover) were measured.

### Perezone quantification

#### Gas chromatography coupled to mass spectrometry

Three sections of each rhizome sample of *A. cordata* were used for perezone quantification. The perezone was extracted three times *via* maceration of 2.0 g (dry weight) of grinded rhizome tissue with 20.0 ml of n-hexane over 3 days. The extracts were then obtained by solvent evaporation in an extraction chamber at room temperature until dry. The extracts were then dissolved in methanol and analyzed by gas chromatography coupled to mass spectrometry. We used an Agilent Technology 6890 gas chromatograph coupled to a MSD 5973 quadrupole mass detector (HP Agilent, Santa Clara, CA, USA) to quantify the perezone using a standard of pure perezone as the reference.

The compounds were separated on an HP-SMS capillary column 30 mm long with a 0.25-mm internal diameter and 0.25-μm film. The carrier gas was helium administered at a linear injection rate of 1.0 ml/min at constant flow. The inlet temperature of the injector was 250 °C, and the temperature of the column was initially 40 °C and increased at 10 °C/min until it reached 250 °C where it was held for 20 min. The mass spectrometer was operated in positive detection mode with an ionization energy of 70 eV. The acquisition was made in selective ion monitoring (SIM), and chromatographic peaks were identified and quantified using target ions appeared (Sánchez-Ramos et al., 2018).

### Rhizospheric soil analysis

To characterize the soil, we collected 60 samples over three localities (20/locality) and collected individual rhizospheric soil samples from the site where each plant grew. For this analysis we took 200 g of dry rhizospheric soil and followed the procedures described by Valencia-Cuevas et al. (2020) quantifying pH and the content of organic matter (O.M.), ammonium (NH_4_), nitrate (NO_3_), phosphate (PO_4_), total nitrogen (N_t_) and total phosphorous (P_t_). For details see the authors (Bremner, 1965; Valencia-Cuevas et al., 2020; Bray & Kurtz, 1945).

### Data analyses

We used a general linear model (GLM) approach to examine whether the soil parameters (pH, O.M., NH_4_, NO_3_, PO_4_, N_t_, P_t_) and the measured macromorphological characteristics (basal diameter, number of branches, total individual height, and shrub cover) influence the perezone production in *A. cordata*. The collection sites were considered as random factors, and pH, O.M., NH_4_, NO_3_, PO_4_, N_t_, P_t_, basal diameter, number of branches, total individual height, and shrub cover as our factors (Table 1). The software used for statistical analysis was STATISTICA 8.0 (Weiß, 2007).

**Table 1:** General lineal model analysis testing the influence of different soil parameters and macromorphological characters on perezone contents in *Acourtia cordata* in three localities at the “Chichinautzin”, Morelos, Mexico.

	*SS*	*F*_*1,46*_	*P*	% Variation
Soil parameter				
pH	2.17	1.20	^n.s.^	
O.M.	17.55	9.75	**	21.2
NH_4_	1.64	0.91	^n.s.^	
NO_3_	1.08	0.60	^n.s.^	
PO_4_	0.39	0.22	^n.s.^	
N_t_	6.83	3.79	*	8.3
P_t_	28.71	15.96	***	34.7
Macromorphological character				
Basal diameter	0.47	0.26	^n.s.^	
Number of branches	0.42	0.23	^n.s.^	
Total individual height	0.60	0.33	^n.s.^	
Shrub cover	0.01	0.00	^n.s.^	
Locality				
Study site	2.34	0.65	^n.s.^	

**Notes:**

n.s. not significant differences.

* *p* < 0.05.

** *p* < 0.01.

*** *p* < 0.005.

### Proteomic analysis

#### Root tissue cleaning

Before protein extraction, each rhizome sample was cleaned according to Wang et al. (2003) with some modifications (Martinez-Esteso et al., 2011). For protein extraction, 4.0 g of tissue was prepared in a mortar and pestle with liquid nitrogen. The tissue was then transferred to 50.0 ml centrifuge tubes, suspended in 10.0 ml of n-hexane, agitated on vortex per 30 s, and centrifuged at 10,000×*g* per 5 min at 4 °C. The supernatant was then discarded, and the pellet was cleaned again 3–4 times until the supernatant was colorless. Next, the pellet was re-suspended in 10.0 ml of a mixture of ethyl acetate:ethanol (1:2) for 1 min and centrifuged 5 min at 4 °C. The wash was repeated followed by discarding the supernatant and re-suspending the sample after the addition of 10.0 ml pure acetone. This procedure includes resuspension, centrifugation, and the discarding of the supernatant. This was repeated during the following steps changing only the resuspension solution to 10% trichloroacetic acid (TCA) in cold acetone, 10% TCA in cold water, and finally, 80% acetone. At each step, the sample was centrifuged at 10,000 × *g* and 4 °C. Finally, the pellet was dried at room temperature and then stored at −80 °C for future usage.

#### Protein extraction

For protein extraction and further protein analysis, all individual rhizome samples were grouped according to their perezone yield using a statistical mean (k-means) test for grouping. Three groups were generated: high, medium, and low producers of perezone (Fig. S3). To obtain protein samples with clear differences for the proteomic analysis, only two groups were analyzed in this study: groups with high and low perezone production.

The cleaned dry powder of tissue (about 250 mg) was homogenized in 2.0 ml microtubes using 0.7 ml extraction buffer containing 0.7 M sucrose, 0.5 M Tris-HCl pH 8.0, 0.1 M HCl, 50 mM EDTA, 1% DTT, 1% polyvinylpolypyrrolidone (PVPP) and a cocktail of protease inhibitors (4-(2-aminoethyl) benzenesulfonyl fluoride, E-64, bestatin, leupeptin, aprotinin and sodium EDTA (Sigma-Aldrich, St. Louis, MO, USA). The homogenate was added with 0.7 ml Tris-saturated phenol pH 8.0 (Sigma, St. Louis, MO, USA). The mixture was vortexed thoroughly for 30 s and incubated with orbital shaking on ice for 1 h. The phenol phase was separated by centrifuging at 10,000 × *g* for 20 min at 4 °C. The upper phenol phase was recovered and pipetted to new 2.0-ml microtubes. The remaining aqueous phase was re-extracted with 0.7 ml Tris-saturated phenol pH 8.0 and 0.7 ml of extraction buffer. Proteins were precipitated from the pooled phenol phases by adding five volumes cold 0.1 M ammonium acetate in methanol, incubating at −20 °C overnight, and collecting by centrifuge at 10,000 × *g* for 10 min at 4 °C. The protein pellet was washed twice with 0.1 M ammonium acetate in methanol and twice with chilled 80% acetone. The pellet was resuspended in 6 M urea. Finally, the protein concentration was determined by RCDC protein assay (BIO-RAD, Madrid, Spain) based on the modified Lowry protein assay (Raghupathi & Diwan, 1994). Protein extracts were also visualized on SDS-PAGE (Fig. S4).

### Liquid protein digestion and protein analysis by ultra-high-performance liquid chromatography coupled with quadrupole time-of-flight

One hundred micrograms of protein extracts were reduced with 5 µl of 0.2 M DTT. The reaction was kept at 37 °C for 60 min. Samples were alkylated with 20 µl of 0.2 M iodoacetamide (IAM) at room temperature for 60 min in the dark. The urea concentration during digestion was reduced 10 times with 25 mM ammonium bicarbonate (AMBIC) which is compatible with trypsin activity. Modified sequencing-grade trypsin was added at a 30:1 protein/enzyme ratio, and samples were digested at 37 °C overnight. Finally, we added trypsin 60:1 protein/enzyme ratio and allowed digestion to proceed for an additional 4–5 h at 37 °C. The tryptic digestion was stopped by acidifying the sample with formic acid. Once digested, each sample was evaporated and resuspended in 0.5% TCA in 5% acetonitrile (ACN) to desalt it. The peptides were passed through a C18 column (Pierce® C18 Spin Columns; Thermo Scientific, Waltham, MA, USA) previously prepared to be activated with 50% ACN buffer and calibrated with a solution of 0.5% TCA in 5% ACN. Once the peptides were inside the column, they were washed with activation buffer and eluted with 70% ACN and 0.1% formic acid; the solvent was then evaporated. Peptides were resuspended in 3% acetonitrile and 0.1% formic acid prior to LC-MS/MS analysis.

The desalted peptide digests were analyzed on an Agilent 6550 iFunnel Q-TOF mass spectrometer (Agilent Technologies, Santa Clara, CA, USA) coupled to an Agilent 1290 UHPLC system. Peptide samples were loaded onto an Agilent AdvanceBio Peptide mapping column (2.1 mm × 250 mm, 2.7 μm particle size, operating at 50 °C) *via* an Infinity Autosampler (Agilent Technologies, Santa Clara, CA, USA) with Buffer A (water, 0.1% formic acid) flowing at 0.4 ml/min. Peptides were eluted into the mass spectrometer using a 140 min linear gradient of 3–40% ACN in 0.1% formic acid. Peptides were introduced to the mass spectrometer from the LC using a Jet Stream source (Agilent Technologies, Santa Clara, CA, USA) operating in positive-ion mode (3,500 V) and high-sensitivity mode. Source parameters employed gas temp (250 °C), drying gas (14.0 l/min), nebulizer (35 psi), sheath gas temp (250 °C), sheath gas flow (11.0 l/min), VCap (3,500 V), fragmentor (360 V), and OCT 1 RF Vpp (750 V). The data were acquired as described Torregrosa-Crespo et al. (2020) with the same Workstation Software and LC/MS Data Acquisition, operating in the same Auto MS/MS mode. For more details, see the authors.

Each MS/MS spectrum was preprocessed with the extraction tool Spectrum Mill Proteomics Workbench (Agilent, Santa Clara, CA, USA) to obtain a peak list and to improve the spectral quality by merging MS/MS spectra with the same precursor (Δm/z < 1.4 Da and chromatographic Δt < 15 s). The reduced dataset was searched against a subset of the NCBInr protein database composed of sequences from the Asteraceae family, and contaminant proteins without taxonomical restrictions was searched in identity mode with the MS/MS search tool of Spectrum Mill Proteomics Workbench. The settings were as follows: trypsin, up to two missed cleavages, carbamidomethylation of cysteines as fixed modifications, oxidation of methionine as a variable, and mass tolerance of 20 ppm for precursor and 50 ppm for product ions. Peptide hits were validated in the peptide mode (FDR < 1.2%) and in protein mode according to the score settings recommended by the manufacturer. At the peptide level, the identification threshold was set at score ≥ 6 and % SPI ≥ 60 (the percentage of the extracted spectrum that is explained by the database search result). In Spectrum Mill, highly confidence identifications were done at the protein level and were considered when two or more peptides were matched. Their summed score is >20; however, the identification relies on highly conserved peptides because the database does not contain any sequence of *Acourtia cordata*; thus, we used all proteins identified through the surrogate-identified peptides for further bioinformatic analysis.

### Quantitative label-free LC-MS analysis

LC-MS analysis without labels used Progenesis QI for proteomics software version 4.1 (NonLinear Dynamics, Newcastle upon Tyne, UK) as recommended by the manufacturer (www.nonlinear.com). The software processed the raw data in two steps as described by Meleady et al. (2012). First, each sample run was subjected to an alignment that involved aligning the data according to the LC retention time of each sample. This allows for any deviation in retention time that gives a tight retention time for all runs in the analysis. The execution of the sample that produced most of the characteristics (*i.e*., peptide ions) was used as the reference run, to which the retention time of all other runs was adjusted; the maximum intensities were then normalized. The Progenesis peptide quantification algorithm calculates the abundance of peptides as the sum of the areas of the peaks within their isotopic limits. Each abundance value is then transformed into a normalized abundance value by applying a global scale factor. The abundance of the proteins was automatically calculated by the Hi-3 as described by Silva et al. (2006) implemented in the Progenesis QI for proteomics.

### Categorization and functional annotation

Proteins with differential abundances were considered upregulated when log2 was 1.0 or greater and downregulated when log2 was −1.0 or less. Differentially expressed proteins were classified by their Gene Ontology (GO) using Blast2GO v5 (https://www.blast2go.com/) (Meleady et al., 2012). Three different GO vocabularies were assigned including biological process, molecular function, and cellular component. A sequence file of FASTA sequences was generated from the group of proteins previously identified and quantified using the NCBI website. Blast2GO was charged with the FASTA file; first, the sequence description was introduced by a BLASTp searching *vs*. NCBInr (e-value cut-off 1 × 10^10^), then the terms GO, IC and Interpro were mapped, followed by sequence annotation (1 × 10^6^ E-value Hit-Filter, cut off 0 Hsp-Hit covering, 55 annotation limit and five GO weight). The analysis of the metabolic pathways in which the identified proteins were classified was performed with the Kyoto Encyclopedia of Genes and Genomes (KEGG) database (http://www.genome.jp/kegg/) (Kanehisa et al., 2017). Finally, we analyzed protein-protein interaction for identified proteins using Cytoscape 3.6.1 software (Shannon et al., 2003). A protein-protein interaction network was obtained from the STRING database; we used the high score corresponding homologous proteins from the *Arabidopsis thaliana* (http://string-db.org/) (Szklarczyk et al., 2017). The interaction network from STRING was visualized in Cytoscape (http://www.cytoscape.org/). Network clustering was carried out as described by Safari-Alighiarloo et al. (2016) with the ClusterONE algorithm, identifying modules with a minimum density of >0.2 and a degree of >0.07. A *p-value* of < 0.05 for a cluster was considered to be a module (Tables S7 and S8).

## Results

### Perezone quantification in the rhizomes of *Acourtia cordata* wild plants

**Figure 1:**
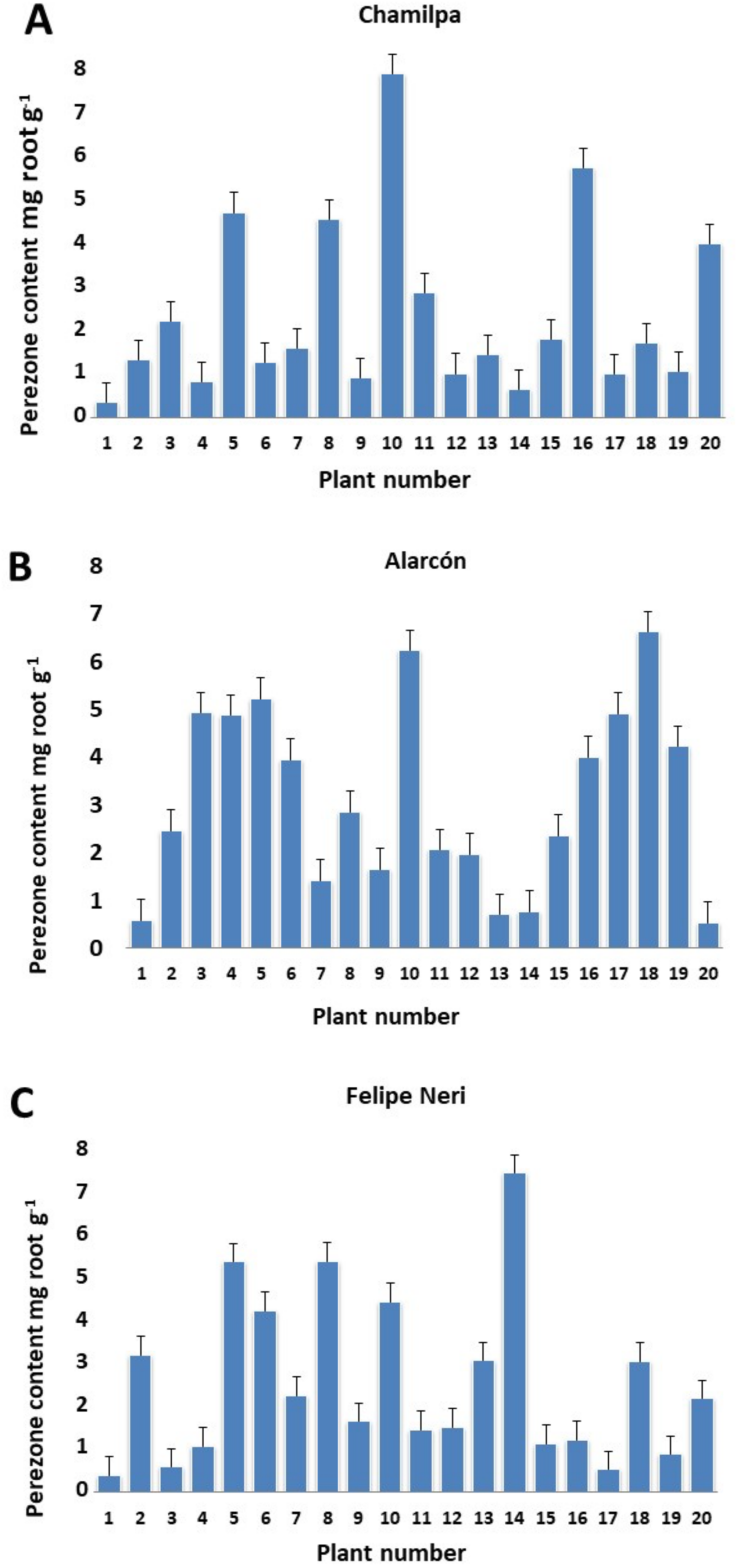
Perezone content in rhizomes of each *Acourtia cordata* plant in the three localities: Chamilpa (A), Alarcón (B) and Felipe Neri (C).

Our results showed significant variation in the production/accumulation of perezone between plant individuals of the same locality (Table S1). The content ranged from 0.329 ± 0.004 to 7.757 ± 0.170 mg g^−1^ of root (Figs. 1A–1C). However, the perezone content did not vary significantly among the three localities studied. We found high producers of perezone that generate between 7.757 ± 0.170 and 4.474 ± 2.9405 × 10^−5^ mg g^−1^ of rhizome, medium producers of perezone that generate between 4.169 ± 0.486 to 2.881 ± 0.060 mg g^−1^, and low producers of perezone that generate between 2.21 ± 0.069 and 0.329 ± 0.004 mg g^−1^ of rhizome (Fig. S3). The identification and quantification of perezone used gas chromatography coupled with mass spectrometry with a retention time between 17.62 and 17.75 min; fragmentation patterns were seen at m/z 166, 191, 205, and 248 (Figs. S5–S7).

### Influence soil parameters and macromorphological characters on perezone contents production

The general linear model (GLM) analysis showed a significant influence of the O.M., N_t_, and P_t_ soil parameters on the perezone contents production (Table 1). GLM analysis showed that P_t_, O.M. and N_t_ had a significantly influenced on perezone content, explaining 34.7%, 21.2% and 8.3% of their variation, respectively. In contrast, there was no relationship between perezone contents and plant morphological characters and collection site (Table 1).

### Identification and functional classification of differentially expressed proteins

The data obtained by mass spectrometry (MS) from each group of *A. cordata* plants were compared with the NCBI database for the Asteraceae family using Spectrum Mill Proteomics Workbench search engine. This approach identified 2,772 proteins (Table S2). We identified 616 differentially expressed proteins when comparing the high production group against the low perezone production group, from which 125 (20.3%) proteins correspond to over-expressed proteins and 491 (79.7%) to repressed proteins. To understand such differences, we performed a further analysis.

Blast2Go software (Conesa & Götz, 2008**) **was used for the automatic assignment of proteins description and could annotate the sequences compared by homology with the database of NCBI. The proteins over-expressed and repressed were analyzed and classified according to their gene ontology (GO) as follows: biological process (GOBP), molecular function (GOMF), and cellular component (GOCC). We note that some proteins were identified and classified in more than one GO category, so the total number of proteins in each graphic may be higher than the initial number (Tables S3 and S4).

### Proteomic comparison between high and low perezone producers

**Figure 2:**
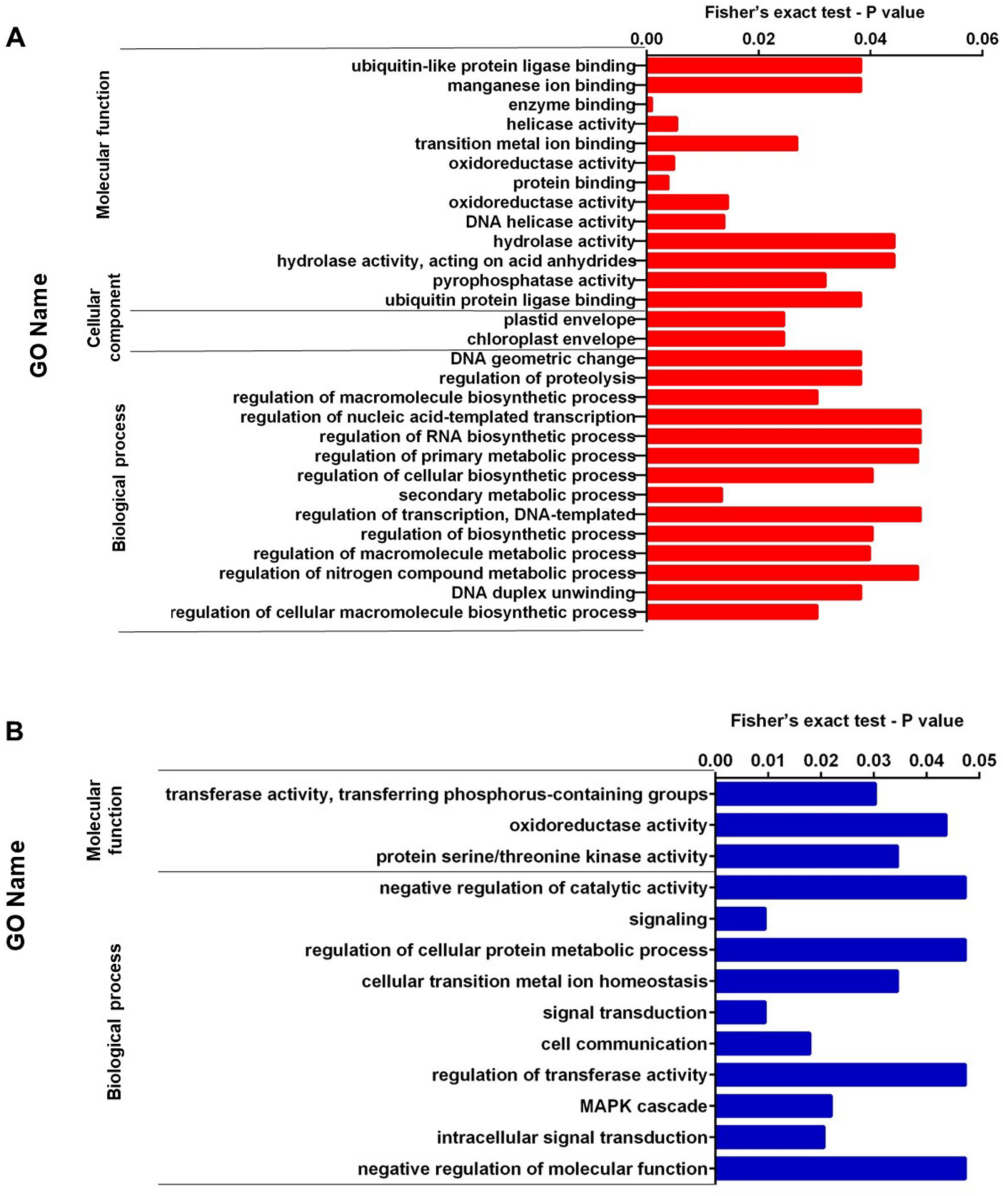
(A) Representation of the set of over-expressed proteins grouped within the GO terms: molecular function, cellular component and biological process. (B) Representation of the set of under-expressed proteins grouped within the GO terms of molecular function and biological process.

**Figure 3:**
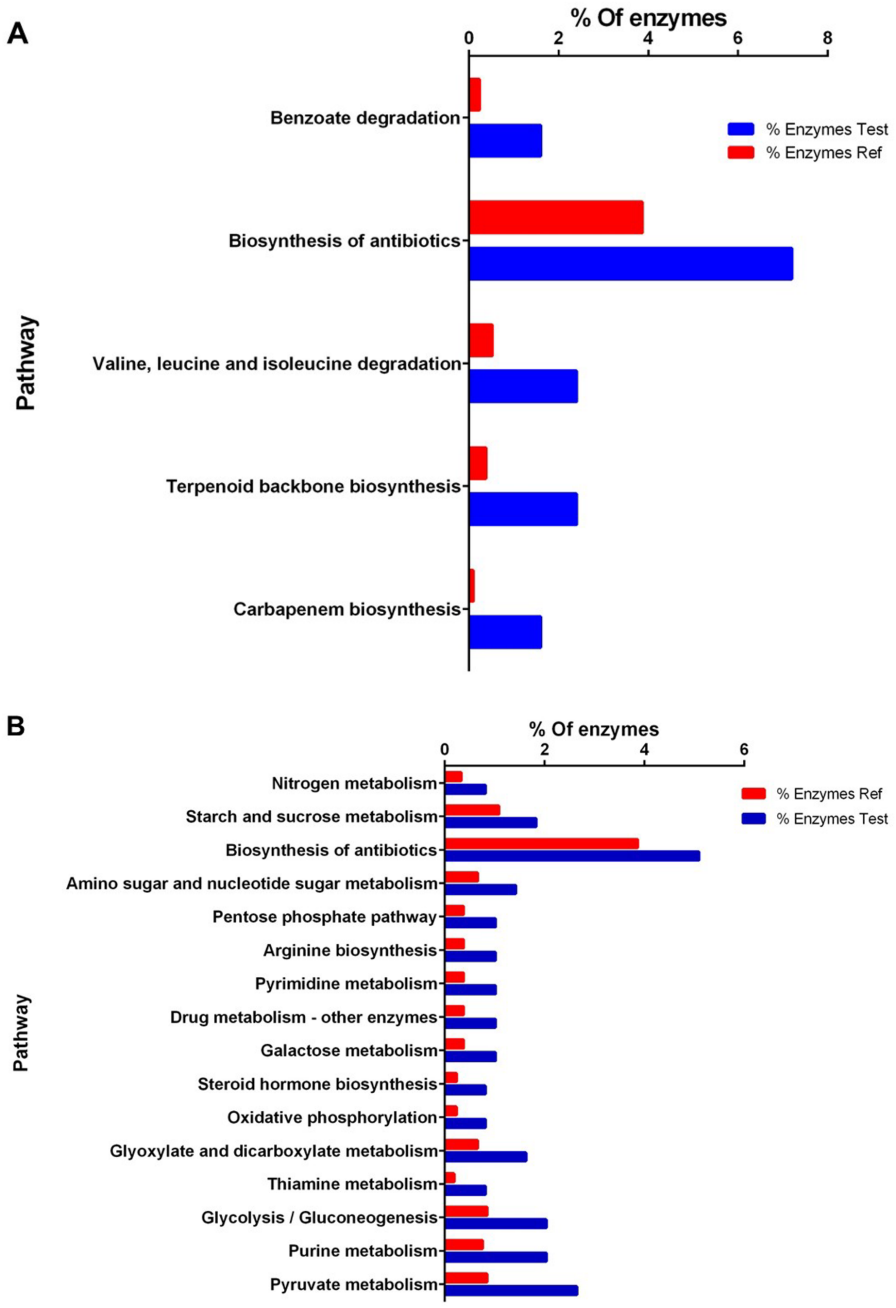
Enrichment analysis of KEGG pathways of differentially expressed proteins between high and low perezone producers. (A) Comparison of the group of over expressed proteins between high and low perezone producers. (B) Comparison of the group of subexpressed proteins between high and low perezone producers.

We performed enrichment analysis of up-regulated and down-regulated proteins. Figures 2A, 2B, 3A, and 3B show the top significantly enriched GO terms and pathways. The most enriched GOBP categories in up-regulated proteins were the secondary metabolic process showing regulation of macromolecule biosynthetic process, DNA duplex unwinding, regulation of proteolysis, and DNA geometric change. These GO terms represent 88% of the GOBP category. The GOCC categories include the chloroplast envelope and plastid envelope; these GO terms represent 4.8% of the GOCC category. Finally, the GOMF category contained enzyme binding, protein binding, oxidoreductase activity, helicase activity, DNA helicase activity, and pyrophosphatase activity; these GO terms represent 60% of the GOMF category (Fig. 2A). In contrast, the enriched GOBP categories in down-regulated proteins were signal transduction, cell communication, MAPK cascade, and regulation of transferase activity; these GO terms represent 11% of the GOBP category. The GOMF categories were transferase activity transferring phosphorus-containing groups, protein serine/threonine kinase activity, and oxidoreductase activity; these GO terms represent 8.5% of the GOMF category (Fig. 2B).

Over-expressed and repressed proteins of both high and low perezone producers were also analyzed by the KEGG. This work used an enrichment analysis based on the Fisher exact test (*p*-value < 0.05) (Table S5 and S6). The enriched up-regulated metabolic pathways *via* KEGG were compared by comparing high and low perezone producers. These included carbapenem biosynthesis, terpenoid backbone biosynthesis, valine, leucine, and isoleucine degradation, biosynthesis of antibiotics, and benzoate degradation (Fig. 3A). The main down-regulated metabolic pathways in KEGG include pyruvate metabolism, purine metabolism, glycolysis/gluconeogenesis, thiamine metabolism, glyoxylate and dicarboxylate metabolism, oxidative phosphorylation, steroid hormone biosynthesis, galactose metabolism, drug metabolism, pyrimidine metabolism, arginine biosynthesis, pentose phosphate pathway, amino sugar and nucleotide sugar metabolism, biosynthesis of antibiotics, starch and sucrose metabolism, and nitrogen metabolism (Fig. 3B). The importance of this analysis is discussed below.

### Protein-protein interaction analysis

We further searched for predicted interactions for the differentially expressed proteins identified by UHPLC-QTOF proteomics in the STRING protein-protein interaction database. We constructed a protein-protein interaction network in Cytoscape-Cluster ONE. The protein network analysis showed that five clusters were created in the up-regulated proteins group (Table S7). The network predicted an interaction among AT1G11660, FPS1, HSC70-1, P5CS2, AT5G47720, MFP2, AIM1, GAPC2, PKT4, PKT3, AT4G13010, ACAT2, AOR, and KAT5 (cluster 1). This group of proteins is involved in processes such as stress response, defense response, biosynthesis of SM, and fatty acid metabolism. An interaction was also observed among PHYA, FUS5, COP8, COP13, FUS12, COP9, and FUS6 (cluster 2). These proteins are involved in processes such as response to abiotic stimulus as well as an interaction among ESP3, At1g80070, MAC3A, MAC3B, CDC5, AT5G46840, MOS4, and AT3G19810 (cluster 3); these proteins are involved in spliceosome process. Finally, there was an interaction between UPF3, DCP2, LBA1, DCP1, and AT2G39260 with AT2G40770, AT1G30680, and SRS2, resulting in clusters 4 and 5, respectively; these proteins are involved in plant development and adaptation.

Nine clusters were created in the down-regulated proteins group (Table S8). The first cluster grouped proteins like LOS1, AT2G45030, RPS11, RPS5A, AT1G67430, RPS5B, AT1G07210, AT3G09630, and AT2G09990. These proteins are involved in the ribosome pathway and organic nitrogen compound biosynthetic processes. The interaction among AT5G51570, VHA-A3, AT3G42050, AT3G28715, VHA-A, and TUF (cluster 2) are vacuolar. An interaction was also observed among proteins such as NUP155, NUP1, NTF2B, EMB3142, and RAN3 (cluster 3) that are specifically involved in nucleocytoplasmic transport. EMB1467, NDHF, MATK, and NAD7 (cluster 4) are proteins that form part of ATP synthesis coupled with electron transport, cellular respiration, photorespiration, response to oxidative stress, as well as ATP synthesis-coupled electron transport. Among the interaction of AT3G53980, AGP30, and AT1G17860 (cluster 5) are proteins involved in processes such as systemic acquired resistance, regulation of root development, regulation of seed dormancy process, and endopeptidase inhibitor activity. The interaction among MDH, GDH2, PGMP, ALDH11A3, PKP-ALPHA, NADP-ME4, HXK2, NADP-ME1, PPC3, CSY2, PMDH1, TPI, GLN1-1, and IDH-V (cluster 6) are proteins involved in processes such as malate metabolic process, response to cold, cellular amino acid metabolic process, response to cadmium ion, response to salt stress, fatty acid biosynthetic process, hexokinase-dependent signaling, programmed cell death, leaf development, and photosynthesis. The interaction between LD, FLK, and AT2G47820 (cluster 7) results in this group of proteins being involved in cell differentiation, flower development, vegetative to reproductive phase transition of meristem, and positive regulation of flower development.

The interactions among AT5G46940, UGD3, and RGP5 (cluster 8) are proteins involved in processes such as ionotropic glutamate receptor activity, carbohydrate metabolic process, plant-type cell wall, biogenesis, and response to salt stress. Finally, the interactions among ABCF4, MSR2, and AT2G46290 (cluster 9) are proteins involved in transport, mannan metabolic process, and formation of cytoplasmic translation initiation complex.

## Discussion

### Influence of edaphic factors on the production of perezone

The perezone content could be considered constant in all three localities as can be deduced from the statistical analysis (Table 1), probably because the three studied localities are in the same natural area with the same geological history with similar climatic and altitudinal conditions (Vega Guzmán, López-García & Manzo Delgado, 2008). They are also associated with vegetation dominated by *Quercus*-*Pinus* forests (Fig. S1) (Jaimes-Viera et al., 2018). Another possibility is that the individuals associated with the three localities studied correspond to the same *A. cordata* population, although localities are several kilometers apart. Therefore, future research could be necessary to study the structure and genetic diversity to clarify whether the individuals of the three studied sites are part of the same population.

The high variation observed in perezone content among individual *A. cordata* wild plants at the same locality (Figs. 1A–1C) can be attributed to possible genotypic differences; nevertheless, environmental factors may also have an important role. Therefore, the genotypic characteristics of each individual plant as well as the variability of the environment could determine the intra-population variation observed in perezone content because it is known that the genotypic variation in wild plants could be a significant contribution to qualitative and quantitative variability of secondary metabolites; in this sense, the interaction with environmental factors strongly influences secondary metabolism in wild plants (Moore et al., 2014; Soltis & Kliebenstein, 2015; Moreira et al., 2019).

The high perezone content among plants of the same locality positively correlates with organic matter, ammonium, total nitrogen, and total phosphorus. In this sense, it could be associated with the role that nitrogen and phosphorus play in processes such as photosynthesis and electron transport as well as the availability of precursor molecules for terpene biosynthesis in species that emit and accumulate terpenes. Ormeño & Fernandez (2012) mentioned that: “Nitrogen could promote terpenoid emissions by increasing electron transport rate and leaf photosynthesis, which provide ATP requirements and carbon substrate availability for isoprene synthesis. Phosphorus is expected to influence terpenoid production since terpenoid precursors (IPP: isopentenyl diphosphate and DMAPP: dimethylallyl pyrophosphate) contain high-energy phosphate bonds and phosphorus is a key component of ATP and NADPH, which are required for photosynthesis and terpenoid synthesis (Ormeño & Fernandez, 2012).

We found a positive and significant relationship between ammonium and perezone content that could be important. The assimilation of ammonium by the plant roots releases hydrogen ions (H+) into the soil, which in turn changes the soil pH leading to an increase in the acidity of the rhizospheric soil (Bolan, Hedley & White, 1991) that could be related to higher perezone content. In addition, it seems that these plants prefer ammonium as a source of nitrogen relative to nitrate perhaps because the assimilation of ammonia is less energetically expensive than that of nitrate (Hachiya & Sakakibara, 2017). Some authors state that the accumulation of SM is strongly affected by environmental factors; thus, plants regulate the type and quantity of SM according to environmental variations (Liu et al., 2015).

### Comparison of the proteome of perezone-producing rhizomes

*A. cordata* is a medicinal plant that has been used as a source of perezone and other sesquiterpenes with several applications of pharmacological importance. However, it is a species without a sequenced and annotated genome. It is a “non-model plant” or an “orphan organism”, and proteomic research into non-model organisms like this is particularly challenging. However, we could still analyze the proteome of the perezone-producing rhizomes thanks to advances in proteomics. Other orphan organisms have been analyzed for secondary metabolism *via* proteomic studies: *Picrorhiza kurroa*, *Podophyllum hexandrum*, *Panax ginseng*, *Catharanthus roseus*, *Withania somnifera*, *Euphorbia kansui*, and *Silybum marianum* (Senthil et al., 2011; Champagne et al., 2012; Bhattacharyya et al., 2012; Gharechahi et al., 2013; Ma et al., 2013; Sud, Chauhan & Tandon, 2014; Zongo et al., 2013; Zhao et al., 2014; Romero-Sandoval, Kolano & Alvarado-Vázquez, 2017).

Previously, we commented that there is a positive relationship between soil nutrients and the perezone content. We explained the possible relationship between these factors and the compound of interest. However, the proteomics approach allowed us to present the results that broadened this perspective. In this sense, we mention that the nutrients in the soil may be found in a way not available for plants, even if the nutrients are in high concentrations. Since the functional annotation GO and KEGG analysis showed metabolic processes and pathways mainly related to the internal lack of nutrients, this hypothesis prompted ideas and possibilities to explain our observed results. Future work will continue to explore the *A. cordata* proteome under different types of stress and relate it to the production of perezone.

In this study, GO annotation analysis revealed that some up-regulated proteins were enriched in processes related to regulating nitrogenous compounds such as DNA and RNA biosynthesis. In addition, the secondary metabolism and the regulation of proteolysis processes were enriched together with the activity of proteins associated with ubiquitination: ubiquitin protein ligase and ubiquitin-like protein ligase play an essential role in the response of plants to nutritional homeostasis (Yates & Sadanandom, 2013; Rojas-Triana et al., 2013). These results indicate that such pathways are mainly related to the response to high nitrogen concentrations in the soil, their low availability and absorption for roots, and the biosynthesis of perezone.

The production of many secondary metabolites agrees with the carbon nutrient balance (CNB) hypothesis prediction (Coviella, 2002); however, many other results do not agree with such predictions (Manninen et al., 2002; Foyer, Ferrario-Méry & Noctor, 2001; Bryant, Chapin & Klein, 1983). Since the CNB hypothesis predicts that the amounts of secondary metabolites in plants crucially depend on carbon and nitrogen availability in their environment, the limited nutrient content, together with unlimited light, leads to growth inhibition and stimulation of carbon-based defense metabolites biosynthesis including tannins and terpenes (Bryant, Chapin & Klein, 1983). Due to these environmental factors, we found over-expressed enzymes in the group of high perezone production that participates in the metabolic pathway of terpenes. These enzymes are Acetyl-CoA C-acetyltransferase, geranyl-diphosphate synthase, and farnesyl diphosphate synthase. All three participate in the biosynthesis of terpenes *via* the mevalonate pathway (MVA) that occurs in the cytosol (Kumari et al., 2013). Farnesyl diphosphate synthase is a key enzyme in the biosynthesis of sesquiterpenes. It catalyzes the consecutive condensations of dimethylallyl diphosphate or geranyl diphosphate with isopentenyl pyrophosphate to produce farnesyl diphosphate (Zhao et al., 2015; Guo, Li & Peng, 2015). This molecule is a precursor of sesquiterpenes and other terpenes such as sterols, brassinosteroids, farnesol, nerolidol, germacrene, and valencene (Lombard & Moreira, 2011); it is likely participating in the biosynthesis of perezone. The sesquiterpenes are mostly volatile compounds with various functions in plants. They are released after damage due to the aerial or underground part of the plant by herbivores or pathogens (Dudareva, Pichersky & Gershenzon, 2004). They are also used as allelopathic agents and have other functions (Kochevenko et al., 2012; Assaeed et al., 2020).

We also found upregulated enzymes such as 2-oxoisovalerate dehydrogenase E2 component, malonate-semialdehyde dehydrogenase, and methylmalonate-semialdehyde dehydrogenase that participate in the valine, leucine, and isoleucine degradation pathway. In plants, branched-chain amino acids are an alternative energy source when the carbohydrate source is limited. These amino acids rapidly degrade under stress scenarios and development (Zhao et al., 2014). Moreover, amino acid catabolism is crucial in respiration in limited light conditions or prolonged darkness (Kochevenko et al., 2012; Araújo et al., 2011; Peng et al., 2015). These data suggest that the degradation of amino acids generates molecules of acetyl-CoA that can be used for the biosynthesis of terpenes. This process could be occurring in *A. cordata* plants with a high perezone yield: an over-expression pathway in terpene biosynthesis was observed along with the pathway of degradation of valine, leucine, and isoleucine.

We also observed some downregulated proteins such as glutamine synthetase participating in nitrogen metabolism. These were mainly seen in nitrate and ammonium assimilation and is also important to the photorespiration process (Lea & Miflin, 1974; Naliwajski & Skłodowska, 2018). We found a serine hydroxymethyltransferase implicated in glyoxylate and dicarboxylate metabolism; in plants, one of the main activities of this enzyme is to catalyze the reversible serine-to-glycine conversion. The one-carbon units mainly result from the activity of this enzyme (Walton & Woolhouse, 1986). Glyoxylate and dicarboxylate metabolism and photorespiration play a vital role in stress conditions, and enzymes such as glutamine synthetase and serine hydroxymethyltransferase are upregulated (Tang et al., 2019); in our study, however, they were downregulated according to other research results published previously. The reason for these results is still unknown, and some studies suggest that it could be related to post-transcriptional regulation (Xu et al., 2018).

In general, we found proteins that were under-expressed in the metabolism of glycolysis and gluconeogenesis, such as pyruvate decarboxylase, triosephosphate isomerase, fructose-bisphosphate aldolase, pyruvate dehydrogenase, phosphoenolpyruvate carboxykinase (GTP), phosphoglucomutase, and pyruvate kinase. One way to generate energy in plants is *via* the degradation of starch and sucrose. Starch is broken down to biosynthesize glucose molecules, which enter the glycolytic pathway (Fernie, Willmitzer & Trethewey, 2002; Bahaji et al., 2014). In summary, the downregulated expression of proteins related to the pentose phosphate pathway, glycolysis, as well as the starch and sucrose metabolism may indicate a lack of carbon skeletons necessary for nitrogen assimilation. These results agree and confirm our assumptions about the lack of internal nutrients and the plant allocation resources to synthesize molecules related to secondary metabolism.

Finally, the differentially expressed proteins were subjected to STRING analysis and visualized using Cytoscape software. The analysis of these proteins indicated that AT1G11660, P5CS2 and FPS1 are directly related and involved in heat stress response, proline biosynthesis, and terpenoid biosynthesis. Proline biosynthesis and accumulation have been reported to be involved in the adaptation of plants to environmental stresses (Hare & Cress, 1997) in addition to acting as an osmo-protective molecule (Meena et al., 2019). Proline is a multifunctional amino acid, and its biosynthesis has been linked to the production of secondary metabolites. In our analysis, the relationship between these proteins is likely linked to high concentrations of perezone and its biosynthesis due to nutritional stress.

In contrast, the biosynthesis of SM including volatile terpenes is related to heat stress. In that sense, Gupta et al. (2014) reported several volatile compounds differentially produced by *Hymenaea courbaril* under heat stress: isoprene, 2-methyl butanenitrile, β-ocimene, and sesquiterpenes (Gupta et al., 2014). Pazouki et al. (2016) demonstrate that heat stress results in a major enhancement of terpenoid emissions in *Solanum lycopersicum* (Bryant, Chapin & Klein, 1983).

The edaphic analysis showed a positive correlation between the concentrations of perezone and the levels of nitrogen, phosphorus, ammonia and organic matter. This relationship is weak and probably not completely linked to perezone biosynthesis because proteins were found overexpressed in the pathway of amino acid degradation. This suggests that the nutrients were not entirely in their available form to *Acourtia cordata* plants. Primary and secondary metabolic pathways did not take the nutrients from the soil to obtain the precursor molecules for the biosynthesis of sesquiterpenes, such as perezone. Indeed, the plants obtained these precursors from alternative routes, such as the degradation of amino acids and not from the photosynthetic pathway. In addition, the degradation of amino acids such as valine, leucine, and isoleucine, is related to low or no light; there could also be low rates of photosynthesis. This last idea agrees with our results because *Acourtia cordata* plants grow under the shade of the trees of the *Quercus*-*Pinus* forest.

## Conclusions

This study correlates edaphic factors such as pH, O.M., N_t_, NH_4_, and P_t_ with the contents of perezone in rhizomes of wild plants of *A. cordata*; it also includes the first proteomic report for *A. cordata* and the first to show a high variation of perezone production in wild plants. Also, it suggests that the genotypic variation could also be playing an important role in the production of perezone.

Moreover, all identified proteins gave a general view of the proteomic profile, specifically of the rhizome of this plant species. Some of these proteins were classified according to their gene ontology in biological processes, cellular components, and molecular function. The KEGG analysis classified proteins in several pathways of basal metabolism, including, glycolysis, gluconeogenesis, metabolism of amino acids/lipids, biosynthesis of SM, or proteins involved in the biosynthetic pathways of terpenes, phenylpropanoids, flavonoids, *etc*.

This work predicted probable interactions between some of the over-expressed proteins. It showed a possible expression of proteins involved in the biosynthesis of compounds, such as sesquiterpenes, together with the expression of related proteins during stress. This work will lead to other research, including further experiments using cloned *A. cordata* plants, to control the response of the genotype on varying pH, nitrogen, and phosphorous fertilizing concentrations, to elucidate the role of these environmental factors on the production/accumulation of perezone. RNA sequencing of high *vs*. low perezone producers could also be another approach.

## Supplemental Information

10.7717/peerj.16136/supp-1 Supplemental Information 1 Rhizomes of *Acourtia cordata* wild plants growing in *Quercus*-*Pinus* forest, were collected at three localities.

10.7717/peerj.16136/supp-2 Supplemental Information 2 Voucher specimen of *Acourtia cordata* plants of the three localities.

10.7717/peerj.16136/supp-3 Supplemental Information 3 Grouping of *Acourtia cordata* plants according with the perezone content in their rhizomes.

10.7717/peerj.16136/supp-4 Supplemental Information 4 Protein profiles of perezone producing roots of *A. cordata*, Molecular Weight Marker (MWM), High 1, 2 and 3 (A1, A2 and A3), Medium 1, 2 and 3 (M1, M2 and M3), Down 1, 2 and 3 (B1, B2 and B3).

10.7717/peerj.16136/supp-5 Supplemental Information 5 Analysis of GC-MS of perezone in the hexane extract of *A.cordata* roots. Identification of the compound in the group of high producers with a RT = 17.75 min and m/z = 166, 191, 205 and 248.

10.7717/peerj.16136/supp-6 Supplemental Information 6 Analysis of GC-MS of perezone in the hexane extract of *A. cordata* roots. Identification of the compound in the group of low producers with a RT = 17.61 min y m/z = 166, 191, 205 and 248.

10.7717/peerj.16136/supp-7 Supplemental Information 7 Figure S7. Analysis of GC-MS of standard perezone. Identification of the compound. RT = 17.61 min y m/z = 166, 191, 205 and 248

10.7717/peerj.16136/supp-8 Supplemental Information 8 Perezone quantification of *A. cordata* rhizomes and data for graphics 1A, 1B and 1C. Plants from 1 to 20 belong to Chamilpa site, Plants from 21 to 40 belong to the Alarcon site, and Plants from 41 to 60 belong to the Felipe Neri site. Replicas 1, 2 and 3 stand for: R1, R2 and R3. Data for the Graphics 1A, 1B and 1C are after the quantification data.

10.7717/peerj.16136/supp-9 Supplemental Information 9 Total identified proteins in *A. cordata* rhizomes.

10.7717/peerj.16136/supp-10 Supplemental Information 10 Gene Ontology (GO) enrichment analyses for overexpressed proteins of *A. cordata*.

10.7717/peerj.16136/supp-11 Supplemental Information 11 Gene Ontology (GO) enrichment analyses for repressed proteins of *A. cordata*.

10.7717/peerj.16136/supp-12 Supplemental Information 12 KEGG enrichment analysis of upregulated proteins of *A. cordata*.

10.7717/peerj.16136/supp-13 Supplemental Information 13 KEGG enrichment analysis of downregulated proteins of *A. cordata*.

10.7717/peerj.16136/supp-14 Supplemental Information 14 List of proteins involved in protein-protein interaction network for upregulated proteins.

10.7717/peerj.16136/supp-15 Supplemental Information 15 List of proteins involved in protein-protein interaction network for downregulated proteins.

## Additional Information and Declarations

### Field Study Permissions

The following information was supplied relating to field study approvals (*i.e*., approving body and any reference numbers): Field collected material was carried out according with the protocols of the Universidad Autónma del Estado de Morelos/Corredor Biológico Chichinautzin.

### Data Availability

The following information was supplied regarding data availability: The raw data are available in the Supplemental Files.

## References

[ref-1] Aghaei K, Komatsu S (2013). Crop and medicinal plants proteomics in response to salt stress. Frontiers in Plant Science.

[ref-2] Alarcon-Aguilar FJ, Roman-Ramos R, Jimenez-Estrada M, Reyes-Chilpa R, Gonzalez-Paredes B, Flores-Saenz JL (1997). Effects of three Mexican medicinal plants (Asteraceae) on blood glucose levels in healthy mice and rabbits. Journal of Ethnopharmacology.

[ref-3] Andrew RL, Peakall R, Wallis IR, Foley WJ (2007). Spatial distribution of defense chemicals and markers and the maintenance of chemical variation. Ecology.

[ref-4] Araújo WL, Tohge T, Ishizaki K, Leaver CJ, Fernie AR (2011). Protein degradation—an alternative respiratory substrate for stressed plants. Trends in Plant Science.

[ref-5] Arellano J, Vazquez F, Villegas T, Hernandez G (1996). Establishment of transformed root cultures of *Perezia cuernavacana* producing the sesquiterpene quinone perezone. Plant Cell Reports.

[ref-6] Armengaud J, Trapp J, Pible O, Geffard O, Chaumot A, Hartmann EM (2014). Non-model organisms, a species endangered by proteogenomics. Journal of Proteomics.

[ref-7] Assaeed A, Elshamy A, El-Gendy AE-N, Dar B, Al-Rowaily S, Abd-ElGawad A (2020). Sesquiterpenes-rich essential oil from above ground parts of *Pulicaria somalensis* exhibited antioxidant activity and allelopathic effect on weeds. Agronomy.

[ref-8] Baginsky S (2008). Plant proteomics: concepts, applications, and novel strategies for data interpretation. Mass Spectrometry Reviews.

[ref-9] Bahaji A, Li J, Sánchez-López AM, Baroja-Fernández E, Muñoz FJ, Ovecka M, Almagro G, Montero M, Ezquer I, Etxeberria E, Pozueta-Romero J (2014). Starch biosynthesis, its regulation and biotechnological approaches to improve crop yields. Biotechnology Advances.

[ref-10] Bassi D, Menossi M, Mattiello L (2018). Nitrogen supply influences photosynthesis establishment along the sugarcane leaf. Scientific Reports.

[ref-11] Bayer EM, Bottrill AR, Walshaw J, Vigouroux M, Naldrett MJ, Thomas CL, Maule AJ (2006). Arabidopsis cell wall proteome defined using multidimensional protein identification technology. Proteomics.

[ref-12] Bhattacharyya D, Sinha R, Ghanta S, Chakraborty A, Hazra S, Chattopadhyay S (2012). Proteins differentially expressed in elicited cell suspension culture of *Podophyllum hexandrum* with enhanced podophyllotoxin content. Proteome Science.

[ref-13] Bolan NS, Hedley MJ, White RE (1991). Processes of soil acidification during nitrogen cycling with emphasis on legume-based pastures. Plant and Soil.

[ref-14] Bray RH, Kurtz LT (1945). Determination of total, organic and available forms of phosphorus in soils. Soil Science.

[ref-15] Bremner JM, Black CA (1965). Inorganic forms of Nitrogen. Methods of Soil Analysis, Part 2, Agronomy Monograph No. 9.

[ref-16] Bryant JP, Chapin FS, Klein DR (1983). Carbon/nutrient balance of boreal plants in relation to vertebrate herbivory. Oikos.

[ref-17] Bryant L, Flatley B, Patole C, Brown GD, Cramer R (2015). Proteomic analysis of *Artemisia annua*—towards elucidating the biosynthetic pathways of the antimalarial pro-drug artemisinin. BMC Plant Biology.

[ref-18] Burgueño-Tapia E, Castillo L, González-Coloma A, Joseph-Nathan P (2008). Antifeedant and phytotoxic activity of the sesquiterpene p-benzoquinone perezone and some of its derivatives. Journal of Chemical Ecology.

[ref-19] Champagne A, Rischer H, Oksman-Caldentey KM, Boutry M (2012). In-depth proteome mining of cultured *Catharanthus roseus* cells identifies candidate proteins involved in the synthesis and transport of secondary metabolites. Proteomics.

[ref-20] Chmielewska K, Rodziewicz P, Swarcewicz B, Sawikowska A, Krajewski P, Marczak L (2016). Analysis of drought-induced proteomic and metabolomic changes in barley (*Hordeum vulgare* L.) leaves and roots unravels some aspects of biochemical mechanisms involved in drought tolerance. Frontiers in Plant Science.

[ref-21] Conesa A, Götz S (2008). Blast2GO: a comprehensive suite for functional analysis in plant genomics. International Journal of Plant Genomics.

[ref-22] Coviella CE (2002). Plant allocation to defensive compounds: interactions between elevated CO2 and nitrogen in transgenic cotton plants. Journal of Experimental Botany.

[ref-23] Di Michele M, Chiatante D, Plomion C, Scippa GS (2006). A proteomic analysis of Spanish broom (*Spartium junceum* L.) root growing on a slope condition. Plant Science.

[ref-24] Dudareva N, Pichersky E, Gershenzon J (2004). Biochemistry of plant volatiles 1. Plant Physiology.

[ref-25] Escobedo-González R, Mendoza P, Nicolás-Vázquez MI, Hernández-Rodríguez M, Martínez J, Miranda Ruvalcaba R, Douglas Kinghorn A, Folk H, Gibons S, Asakawa Y (2022). A time line of perezone, the first isolated secondary metabolite in the new world, covering from 1852 to 2020. Progress in the Chemistry of Organic Natural Products 116.

[ref-26] Fang X, Chen J, Dai L, Ma H, Zhang H, Yang J, Wang F, Ch Yan (2015). Proteomic dissection of plant responses to various pathogens. Proteomics.

[ref-27] Fernandez-Garcia N, Hernandez M, Casado-Vela J, Bru R, Elortza F, Hedden P, Olmos E (2011). Changes to the proteome and targeted metabolites of xylem sap in *Brassica oleracea* in response to salt stress. Plant, Cell & Environment.

[ref-28] Fernie AR, Willmitzer L, Trethewey RN (2002). Sucrose to starch: a transition in molecular plant physiology. Trends in Plant Science.

[ref-29] Filippi A, Braidot E, Petrussa E, Fabro M, Vuerich M, Boscutti F (2021). Plant growth shapes the effects of elevation on the content and variability of flavonoids subalpine bilberry stands. Plant Biology.

[ref-30] Flores y Troncoso, de Asís F (1982). Historia de la medicina en México desde la época de los indios hasta el presente.

[ref-31] Foyer CH, Ferrario-Méry S, Noctor G (2001). Interactions between carbon and nitrogen metabolism. Plant Nitrogen.

[ref-32] Garcia X, Alcantara-Sarabia G, Cartas-Heredia L, Gijon E (1995). Actions of perezone on rat smooth muscle. General Pharmacology: The Vascular System.

[ref-33] Garcia de la Garma J, Fernandez-Garcia N, Bardisi E, Pallol B, Asensio-Rubio JS, Bru R, Olmos E (2015). New insights into plant salt acclimation: the roles of vesicle trafficking and reactive oxygen species signaling in mitochondria and the endomembrane system. New Phytologist.

[ref-34] García-Méndez MC, Encarnación-Guevara SM, Alvarez L, Marquina S, Arellano-García J (2016). Análisis de la producción de perezona entre diferentes raíces de plantas silvestres de la especie *Acourtia hebeclada DC*. Revista Latinoamericana de Química.

[ref-35] Gharechahi J, Khalili M, Hasanloo T, Salekdeh GH (2013). An integrated proteomic approach to decipher the effect of methyl jasmonate elicitation on the proteome of *Silybum marianum* L. hairy roots. Plant Phisiology and Biochemestry.

[ref-36] Ghosh D, Xu J (2014). Abiotic stress responses in plant roots: a proteomics perspective. Frontiers in Plant Science.

[ref-39] Gómez-Serrano G, Cristiani-Urbina E, Villegas-Garrido TL (2010). Establecimiento de protocolos para la propagación in vitro de plantas de *Acourtia cordata* (Cerv.) Turner (Compositae), colectadas en la Sierra de Guadalupe. Polibotánica.

[ref-40] Gómez-Serrano G, Cristiani-Urbina E, Villegas-Garrido TL (2012). Time-dependent perezone production in different culture systems of *Acourtia cordata*. Central European Journal of Biology.

[ref-37] Guo D, Li HL, Peng SQ (2015). Structure conservation and differential expression of farnesyl diphosphate synthase genes in euphorbiaceous plants. International Journal of Molecular Sciences.

[ref-38] Gupta D, Eldakak M, Rohila JS, Basu C (2014). Biochemical analysis of ‘kerosene tree’ *Hymenaea courbaril* L. under heat stress. Plant Signaling & Behavior.

[ref-41] Hachiya T, Sakakibara H (2017). Interactions between nitrate and ammonium in their uptake, allocation, assimilation, and signaling in plants. Journal of Experimental Botany.

[ref-42] Hamilton JG, Zangerl AR, DeLucia EH, Berenbaum MR (2001). The carbon-nutrient balance hypothesis: its rise and fall. Ecology Letters.

[ref-43] Hare PD, Cress WA (1997). Metabolic implications of stress-induced proline accumulation in plants. Plant Growth Regulation.

[ref-44] Jaimes-Viera MC, Martin Del Pozzo AL, Layer PW, Benowitz JA, Nieto-Torres A (2018). Timing the evolution of a monogenetic volcanic field: Sierra Chichinautzin, Central Mexico. Journal of Volcanology and Geothermal Research.

[ref-45] Joseph-Nathan P, González MP, Rodríguez VM (1972). Terpenoids of *Perezia hebeclada*. Phytochemistry.

[ref-46] Joseph-Nathan P, Santillan RL (1989). The chemistry of perezone and its consequences. Studies in Natural Products Chemistry.

[ref-47] Kanehisa M, Furumichi M, Tanabe M, Sato Y, Morishima K (2017). KEGG: new perspectives on genomes, pathways, diseases and drugs. Nucleic Acids Research.

[ref-48] Kochevenko A, Araújo WL, Maloney GS, Tieman DM, Do PT, Taylor MG, Klee HJ, Fernie AR (2012). Catabolism of branched chain amino acids supports respiration but not volatile synthesis in tomato fruits. Molecular Plant.

[ref-49] Koricheva J, Barton KE (2012). Temporal changes in plant secondary metabolite production. The Ecology of Plant Secondary Metabolites.

[ref-50] Kumari S, Priya P, Misra G, Yadav G (2013). Structural and biochemical perspectives in plant isoprenoid biosynthesis. Phytochemistry Reviews.

[ref-51] Lattanzio V, Cardinali A, Ruta C, Fortunato IM, Lattanzio VMT, Linsalata V, Cicco N (2009). Relationship of secondary metabolism to growth in oregano (*Origanum vulgare* L.) shoot cultures under nutritional stress. Environmental and Experimental Botany.

[ref-52] Lea PJ, Miflin BJ (1974). Alternative route for nitrogen assimilation in higher plants. Nature.

[ref-53] Liu W, Liu J, Yin D, Zhao X (2015). Influence of ecological factors on the production of active substances in the anti-cancer plant *Sinopodophyllum hexandrum* (Royle) T.S. Ying. PLOS ONE.

[ref-54] Lombard J, Moreira D (2011). Origins and early evolution of the mevalonate pathway of isoprenoid biosynthesis in the three domains of life. Molecular Biology and Evolution.

[ref-55] Ma R, Sun L, Chen X, Jiang R, Sun H, Zhao D (2013). Proteomic changes in different growth periods of ginseng roots. Plant Physiology and Biochemistry.

[ref-56] Manninen AM, Utriainen J, Holopainen T, Kainulainen P (2002). Terpenoids in the wood of Scots pine and Norway spruce seedlings exposed to ozone at different nitrogen availability. Canadian Journal of Forest Research.

[ref-57] Martinez-Esteso MJ, Sellés-Marchart S, Vera-Urbina JC, Pedreño MA, Bru-Martinez R (2011). DIGE analysis of proteome changes accompanying large resveratrol production by grapevine (*Vitis vinifera* cv. Gamay) cell cultures in response to methyl-β-cyclodextrin and methyl jasmonate elicitors. Journal of Proteomics.

[ref-58] Martinez-Esteso MJ, Vilella-Anton MT, Selles-Marchart S, Martinez-Marquez A, Botta-Catala A, Piñol-Dastis R, Bru-Martínez R (2016). A DIGE proteomic analysis of wheat flag leaf treated with TERRA-SORB® foliar, a free amino acid high content biostimulant. Journal of Integrated OMICS.

[ref-59] Martínez-Esteso MJ, Casado-Vela J, Sellés-Marchart S, Pedreño MA, Bru-Martínez R (2014). Differential plant proteome analysis by isobaric tags for relative and absolute quantitation (iTRAQ). Methods in Molecular Biology.

[ref-60] Martínez-Esteso MJ, Martínez-Márquez A, Sellés-Marchart S, Morante-Carriel JA, Bru-Martínez R (2015). The role of proteomics in progressing insights into plant secondary metabolism. Frontiers in Plant Science.

[ref-61] Martínez-Márquez A, Morante-Carriel J, Sellés-Marchart S, Martínez-Esteso MJ, Pineda-Lucas JL, Luque I, Bru-Martínez R (2013). Development and validation of MRM methods to quantify protein isoforms of polyphenol oxidase in loquat fruits. Journal of Proteome Research.

[ref-62] Meena M, Divyanshu K, Kumar S, Swapnil P, Zehra A, Shukla V, Yadav M, Upadhyay RS (2019). Regulation of L-proline biosynthesis, signal transduction, transport, accumulation and its vital role in plants during variable environmental conditions. Heliyon.

[ref-63] Meleady P, Gallagher M, Clarke C, Henry M, Sanchez N, Barron N, Clynes M (2012). Impact of miR-7 over-expression on the proteome of Chinese hamster ovary cells. Journal of Biotechnology.

[ref-64] Mohamad ZA, Chokchaichamnankit D, Bhinija K, Paricharttanakul NM, Svasti J, Huehne PS, Srisomsap C (2011). Proteomic analysis of Chinese kale (*B. alboglabra*) leaves during growth. Journal of Integrated OMICS.

[ref-65] Moore BD, Andrew RL, Carsten K, Foley WJ (2014). Explaining intraspecific diversity in plant secondary metabolites in an ecological context. New Phytologist.

[ref-66] Moore BD, Foley WJ (2005). Tree use by koalas in a chemically complex landscape. Nature.

[ref-67] Moreira X, Abdala-Roberts L, Nell CS, Vázquez-González C, Pratt JD, Keefover-Ring K, Mooney KA (2019). Sexual and genotypic variation in terpene quantitative and qualitative profiles in the dioecious shrub *Baccharis salicifolia*. Scientific Reports.

[ref-68] Moreira X, Castagneyrol B, Abdala-Roberts L, Berny-Mier y Teran JC, Timmermans BGH, Briun HH, Covelo F, Glauser G, Rasmann S, Tack AJM (2018). Latitudinal variation in plant chemical defences drives latitudinal patterns of leaf herbivory. Ecography.

[ref-69] Naliwajski MR, Skłodowska M (2018). The relationship between carbon and nitrogen metabolism in cucumber leaves acclimated to salt stress. PeerJ.

[ref-71] Oldham JT, Hincapie M, Rejtar T, Wall PK, Carlson JE, Lee-Parsons CWT (2010). Shotgun proteomic analysis of yeast-elicited California poppy (*Eschscholzia californica*) suspension cultures producing enhanced levels of benzophenanthridine alkaloids. Journal of Proteome Research.

[ref-72] Ormeño E, Fernandez C (2012). Effect of soil nutrient on production and diversity of volatile terpenoids from plants. Current Bioactive Compounds.

[ref-73] Pazouki L, Kanagendran A, Li S, Kännaste A, Rajabi Memari H, Bichele R, Niinemets U (2016). Mono- and sesquiterpene release from tomato (*Solanum lycopersicum*) leaves upon mild and severe heat stress and through recovery: from gene expression to emission responses. Environmental and Experimental Botany.

[ref-74] Peng C, Uygun S, Shiu SH, Last RL (2015). The impact of the branched-chain ketoacid dehydrogenase complex on amino acid homeostasis in Arabidopsis. Plant Physiology.

[ref-75] Peña DL, Izaguirre R, Baños G, Viveros M, Enriquez RG, Fernandez JM (2001). Effect of perezone, aminoperezone and their corresponding isomers isoperezone and isoaminoperezone upon in vitro platelet aggregation. Phytomedicine.

[ref-76] Pradhan J, Sahoo SK, Lalotra S, Sarma RS (2017). Positive impact of abiotic stress on medicinal and aromatic plants. International Journal of Plant Sciences.

[ref-77] Raghupathi RN, Diwan AM (1994). A protocol for protein estimation that gives a nearly constant color yield with simple proteins and nullifies the effects of four known interfering agents: microestimation of peptide groups. Analytical Biochemistry.

[ref-78] Ramakrishna A, Ravishankar GA (2011). Influence of abiotic stress signals on secondary metabolites in plants. Plant Signaling & Behavior.

[ref-79] Rojas-Triana M, Bustos R, Espinosa-Ruiz A, Prat S, Paz-Ares J, Rubio V (2013). Roles of ubiquitination in the control of phosphate starvation responses in plants F. Journal of Integrative Plant Biology.

[ref-80] Romero-Sandoval EA, Kolano AL, Alvarado-Vázquez PA (2017). Cannabis and cannabinoids for chronic pain. Current Rheumatology Reports.

[ref-89] Sánchez-Ramos M, Marquina-Bahena S, Romero-Estrada A, Bernabé-Antonio A, Cruz-Sosa F, Gonzálesssz-Christen J, Acevedo-Fernández JJ, Perea-Arango I, Alvarez L (2018). Establishment and phytochemical analysis of a callus culture from *Ageratina pichinchensis* (Asteraceae) and its anti-inflammatory activity. Molecules.

[ref-81] Safari-Alighiarloo N, Rezaei-Tavirani M, Taghizadeh M, Tabatabaei SM, Namaki S (2016). Network-based analysis of differentially expressed genes in cerebrospinal fluid (CSF) and blood reveals new candidate genes for multiple sclerosis. PeerJ.

[ref-82] Senthil K, Karunanithi N, Kim GS, Nagappan A (2011). Proteome analysis of in vitro and in vivo root tissue of *Withania somnifera*. African Journal of Biotechnology.

[ref-83] Sergeant K, Renaut J (2010). Plant biotic stress and proteomics. Current Proteomics.

[ref-84] Shannon P, Markiel A, Ozier O, Baliga NS, Wang JT, Ramage D, Amin N, Schwikowski B, Ideker T (2003). Cytoscape: a software environment for integrated models of biomolecular interaction networks. Genome Research.

[ref-85] Silva JC, Gorenstein MV, Li GZ, Geromanos SJ (2006). Absolute quantification of proteins by LCMSE: a virtue of parallel MS acquisition. Molecular & Cellular Proteomics.

[ref-86] Soltis NE, Kliebenstein DJ (2015). Natural variation of plant metabolism: genetic mechanisms, interpretive caveats, and evolutionary and mechanistic insights. Plant Physiology.

[ref-87] Sud A, Chauhan RS, Tandon C (2014). Mass spectrometric analysis of differentially expressed proteins in an endangered medicinal herb, *Picrorhiza kurroa*. BioMed Research International.

[ref-88] Szklarczyk D, Morris JH, Cook H, Kuhn M, Wyder S, Simonovic M, Santos A, Doncheva NT, Roth A, Bork P, Jensen LJ, Mering CV (2017). The STRING database in 2017: quality-controlled protein-protein association networks, made broadly accessible. Nucleic Acids Research.

[ref-90] Takáč T, Pechan T, Šamaj J (2011). Differential proteomics of plant development. Journal of Proteomics.

[ref-91] Tang J, Sun Z, Chen Q, Damaris NR, Lu B, Hu Z (2019). Nitrogen fertilizer induced alterations in the root proteome of two rice cultivars. International Journal of Molecular Sciences.

[ref-92] Tellez JF, Carvajal K, Cruz D, Cárabez A, Chávez E (1999). Effect of perezone on arrhythmias and markers of cell injury during reperfusion in the anesthetized rat. Life Sciences.

[ref-93] Tian L, Peel GJ, Lei Z, Aziz N, Dai X, He J, Watson B, Zhao PX, Summer LW, Dixon RA (2009). Transcript and proteomic analysis of developing white lupin (*Lupinus albus* L.) roots. BMC Plant Biology.

[ref-94] Torregrosa-Crespo J, Pire C, Richardson DJ, Martínez-Espinosa RM (2020). Exploring the molecular machinery of denitrification in *Haloferax mediterranei* through proteomics. Frontiers in Microbiology.

[ref-95] Trupiano D, Rocco M, Renzone G, Scaloni A, Viscosi V, Chiatante D, Scippa GS (2012). The proteome of *Populus nigra* woody root: response to bending. Annals of Botany.

[ref-96] Valencia-Cuevas L, Rodríguez-Domínguez A, Mussali-Galante P, Ramos-Quintana F, Tovar-Sánchez E (2020). Influence of edaphic factors along an altitudinal gradient on a litter arthropod community in an *Abies*-*Quercus* forest in Mexico. Acta Oecologica.

[ref-97] Vega Guzmán A, López-García J, Manzo Delgado LL (2008). Análisis espectral y visual de vegetación y uso de suelo con imágenes Landsat ETM+ con apoyo de fotografías aéreas digitales en el Corredor Biológico Chichinautzin, Morelos, México. Investigaciones Geográficas. Boletín del Instituto de Geografía.

[ref-98] Villaseñor JL (2016). Catálogo de las plantas vasculares nativas de México. Revista Mexicana de Biodiversidad.

[ref-99] Walton NJ, Woolhouse HW (1986). Enzymes of serine and glycine metabolism in leaves and non-photosynthetic tissues of *Pisum sativum* L. Planta.

[ref-100] Wang W, Scali M, Vignani R, Spadafora A, Sensi E, Mazzuca S, Cresti M (2003). Protein extraction for two-dimensional electrophoresis from olive leaf, a plant tissue containing high levels of interfering compounds. Electrophoresis.

[ref-101] Weiß CH (2007). StatSoft, Inc., Tulsa, OK.: STATISTICA, version 8. AStA Advances in Statistical Analysis.

[ref-102] Xu Y, Zeng X, Wu J, Zhang F, Li C, Jiang J, Wang Y, Sun W (2018). iTRAQ-based quantitative proteome revealed metabolic changes in winter turnip rape (*Brassica rapa* l.) under cold stress. International Journal of Molecular Sciences.

[ref-103] Yates G, Sadanandom A (2013). Ubiquitination in plant nutrient utilization. Frontiers in Plant Science.

[ref-105] Zhao YJ, Chen X, Zhang M, Su P, Liu YJ, Tong YR, Wang XJ, Huang LQ, Gao W (2015). Molecular cloning and characterization of farnesyl pyrophosphate synthase from Tripterygium wilfordii. PLOS ONE.

[ref-106] Zhao X, Si J, Miao Y, Peng Y, Wang L, Cai X (2014). Comparative proteomics of *Euphorbia kansui Liou* milky sap at two different developmental stages. Plant Physiology and Biochemistry.

[ref-70] Zongo F, Ribuot C, Boumendjel A, Guissou I (2013). Botany, traditional uses, phytochemistry and pharmacology of *Waltheria indica* L. (syn. *Waltheria americana*): A review. Journal of Ethnopharmacology.

