# Supplementary material for: High variability of perezone content in rhizomes of Acourtia cordata wild plants, environmental factors related, and proteomic analysis
Source: PeerJ. 2023 Nov 15;11:e16136. doi: 10.7717/peerj.16136 (PMC10656900; doi:10.7717/peerj.16136)
Supplement: Supplemental Information 1 [file peerj-11-16136-s001.pdf]

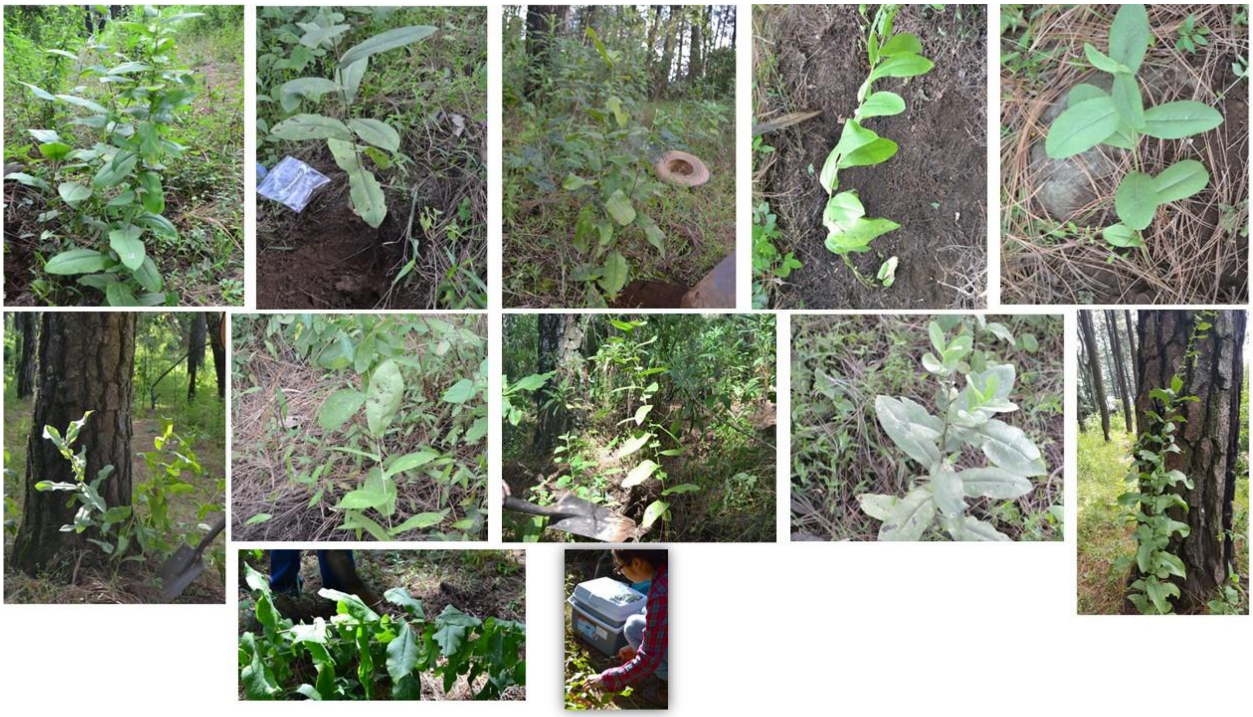

Figure S1. Rhizomes of *Acourtia cordata* wild plants growing in *Quercus*-*Pinus* forest, were collected at three localities.
