# Supplementary figures and images for: High variability of perezone content in rhizomes of Acourtia cordata wild plants, environmental factors related, and proteomic analysis
Source: PeerJ. 2023 Nov 15;11:e16136. doi: 10.7717/peerj.16136 (PMC10656900; doi:10.7717/peerj.16136)

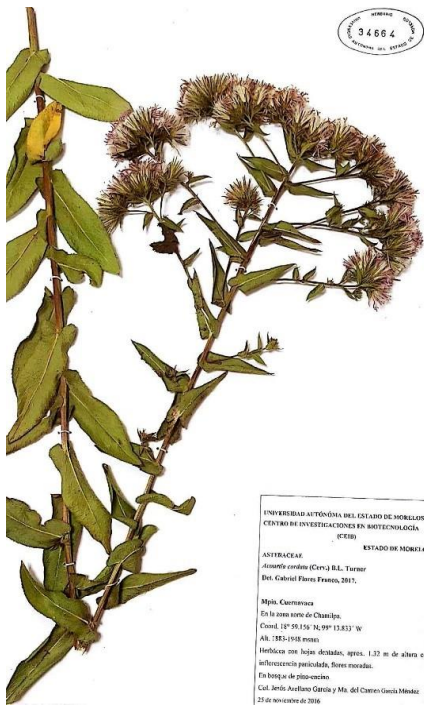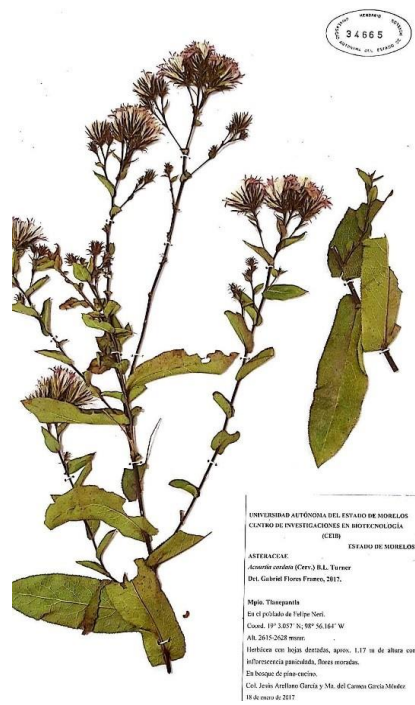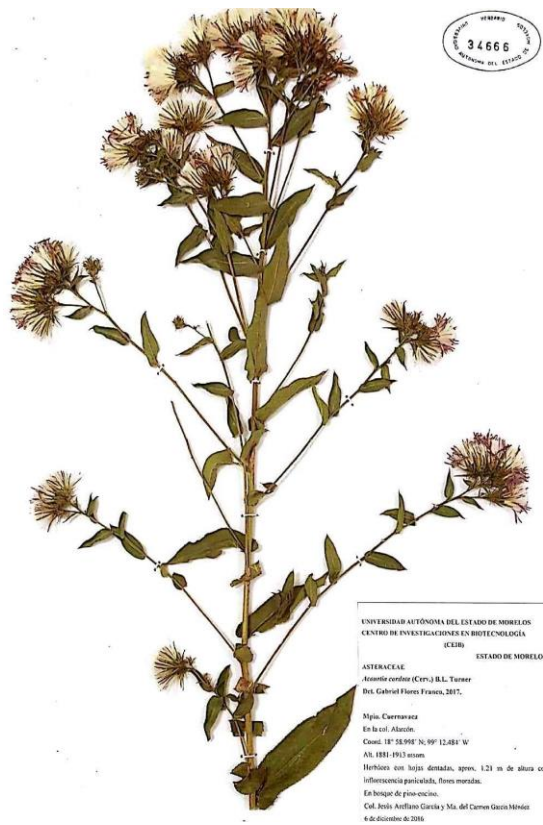

Supplement: Supplemental Information 2 [file peerj-11-16136-s002.pdf]

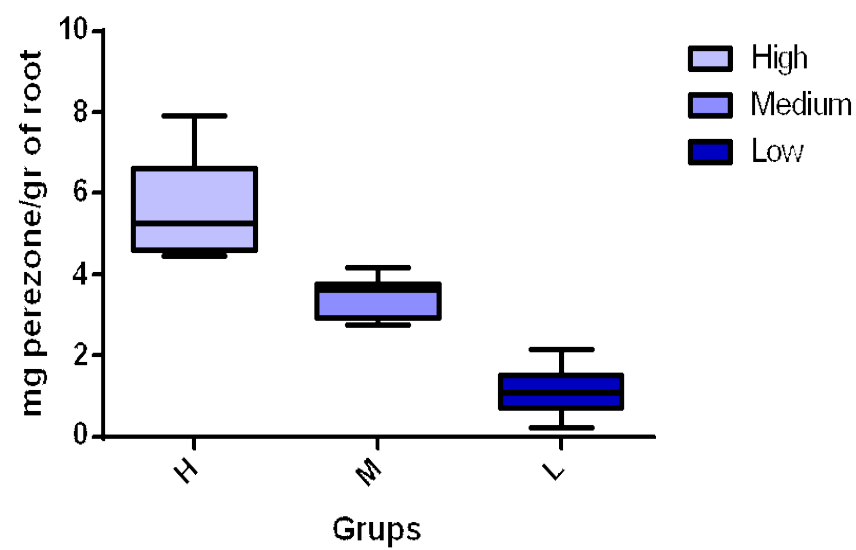

Supplement: Supplemental Information 3 [file peerj-11-16136-s003.pdf]

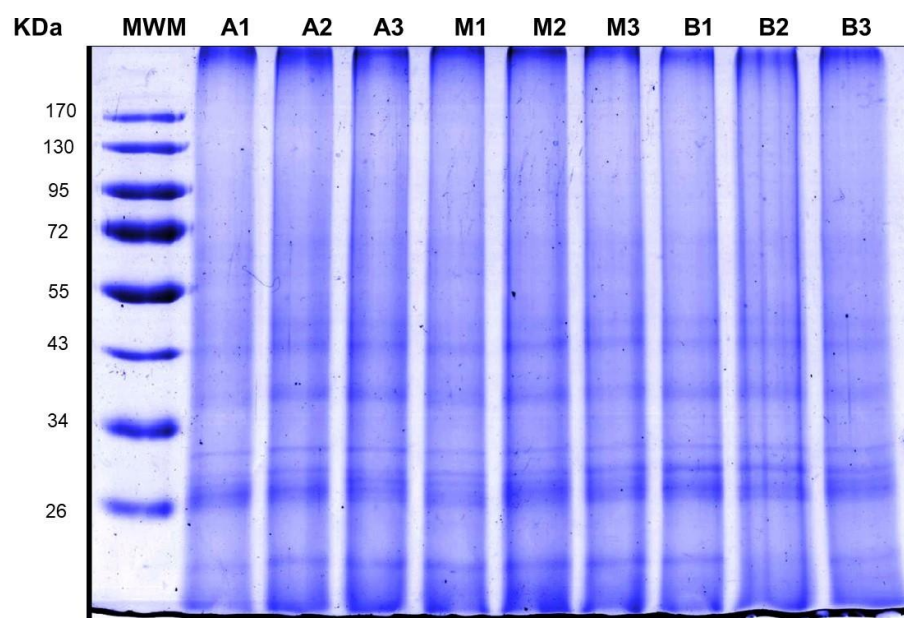

Supplement: Supplemental Information 4 [file peerj-11-16136-s004.pdf]

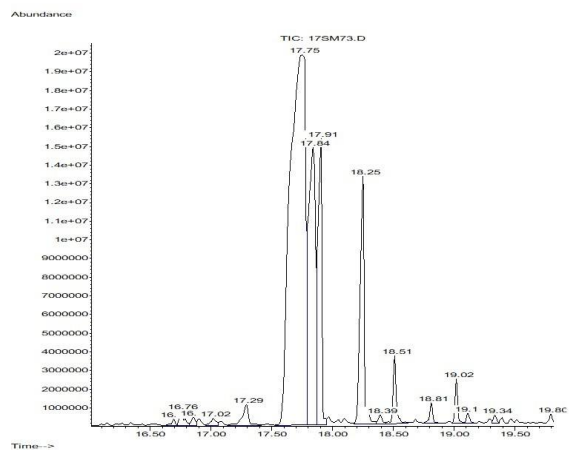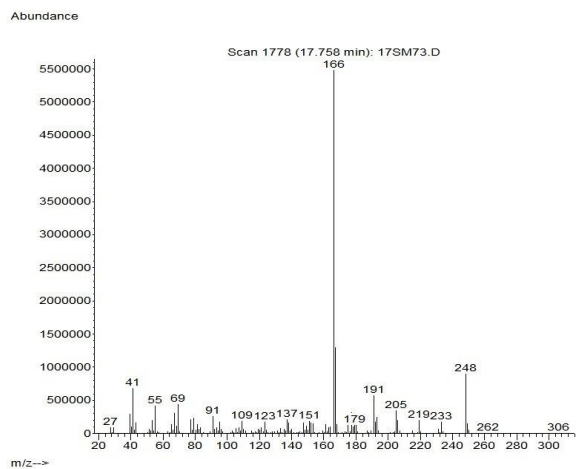

Supplement: Supplemental Information 5 — Identification of the compound in the group of high producers with a RT = 17.75 min and m/z = 166, 191, 205 and 248. [file peerj-11-16136-s005.pdf]

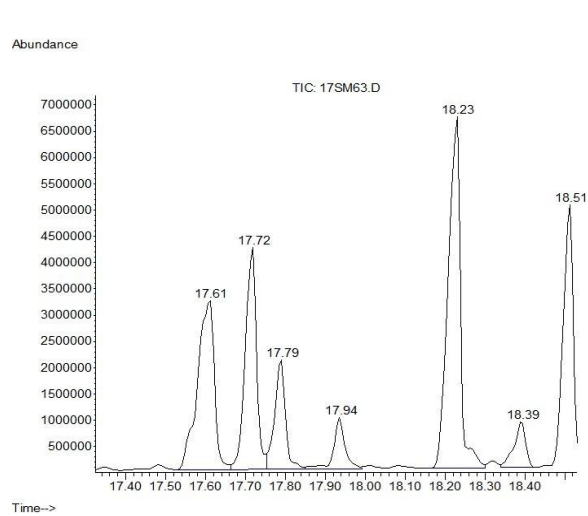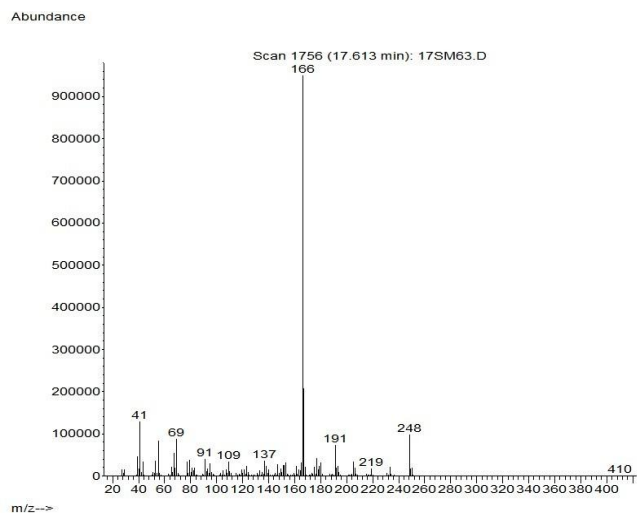

Supplement: Supplemental Information 6 — Identification of the compound in the group of low producers with a RT = 17.61 min y m/z = 166, 191, 205 and 248. [file peerj-11-16136-s006.pdf]

Abundance

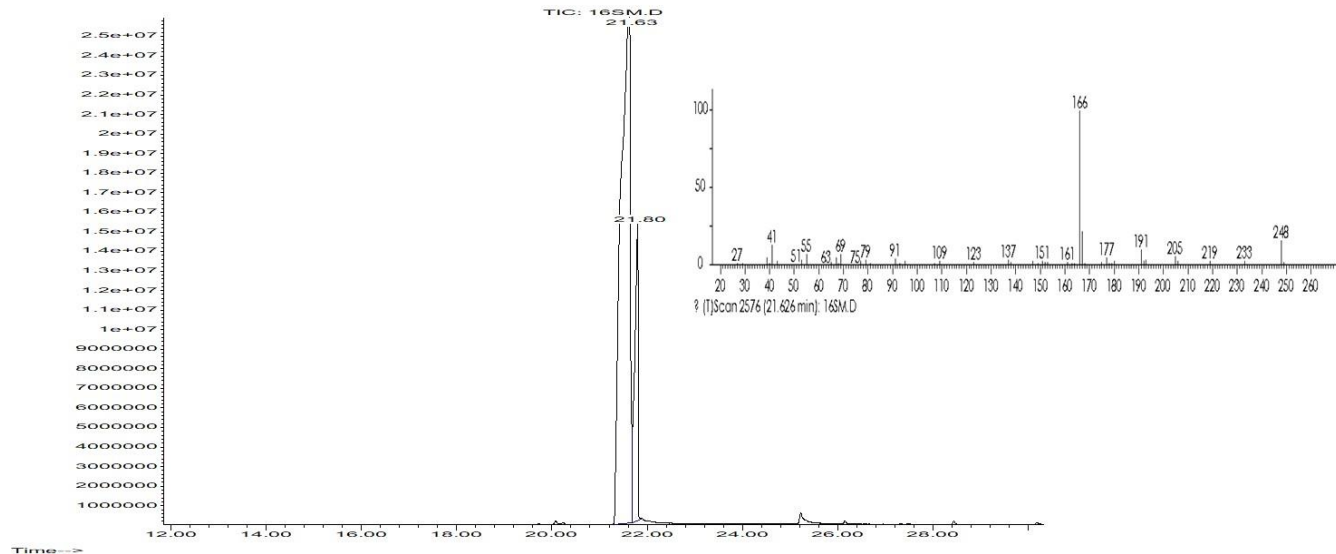

Supplement: Supplemental Information 7 — Analysis of GC-MS of standard perezone. Identification of the compound. RT = 17.61 min y m/z = 166, 191, 205 and 248 [file peerj-11-16136-s007.pdf]
